# Neuro Postural Optimization Neuromodulation Treatment of Radio Electric Asymmetric Conveyer Technology on Stress and Quality of Life in Institutionalized Children in a Capital City of the Brazilian Amazon

**DOI:** 10.7759/cureus.26550

**Published:** 2022-07-04

**Authors:** Ana V Gonçalves de Oliveira Cruz, Rebeca Góes Gonçalves, Lucas Nunes, João Douglas Quaresma de Oliveira, Ester S Lima Monteiro, Italo Soares Eneias, Taynara C Guilherme Lima, Larissa Duarte Ferreira, Erick Souza Neri, José L da Cunha Pena, Anelli M Célis de Cárdenas, Maria I Côrtes Volpe, Maria V Filgueiras de Assis Melo, Arianna Rinaldi, Ana R Pinheiro Barcessat

**Affiliations:** 1 Institutional Program for Scientific Initiation Scholarships (PIBIC), Federal University of Amapá (UNIFAP), Macapá, BRA; 2 Faculty of Nursing Department of Biological and Health Sciences (DCBS), Federal University of Amapá (UNIFAP), Macapá, BRA; 3 Nucleus of Studies in Adaptive Neuro Psycho Physio Pathology (NPNPFPA), Federal University of Amapá (UNIFAP), Macapá, BRA; 4 Institutional Scholarship Program for University Extension (PIBEX), Federal University of Amapá (UNIFAP), Macapá, BRA; 5 Scientific Initiation Scholarship-Affirmative Actions (PROBIC-AF), Federal University of Amapá (UNIFAP), Macapá, BRA; 6 Faculty of Medicine Department of Biological and Health Sciences (DCBS), Federal University of Amapá (UNIFAP), Macapá, BRA; 7 Postgraduate Program in Health Sciences (PPGCS), Federal University of Amapá (UNIFAP), Macapá, BRA; 8 Research Department, Rinaldi Fontani Foundation, Florence, ITA; 9 Department of Adaptive Neuro Psycho Physio Pathology and Neuro Psycho Physical Optimization, Rinaldi Fontani Institute, Florence, ITA; 10 Department of Biomedical Sciences, University of Sassari, Sassari, ITA

**Keywords:** fluctuating asymmetry, reac, quality of life improvement, institutionalized children, quality of life, functional dysmetria, radioelectric asymmetric conveyer, neuromodulation, neuro postural optimization

## Abstract

Background

The deviation from perfect bilateral symmetry is defined as fluctuating asymmetry (FA) and is a common phenomenon among living organisms. This deviation from perfection is thought to reflect the environmental pressures experienced during development and, therefore, the FA represents an epigenetic measure of the environmental stress, which affects all living beings from conception, progressively affecting all aspects of life.

Rinaldi and Fontani hypothesized that the FA morpho-functional changes are originated by an adaptive motor behavior determined by functional alterations in the cerebellum and neural circuits, not caused by a lesion, but induced by the experienced environmental stress. They identified in the asymmetric activation of symmetrical muscle groups, detectable even in healthy subjects, the expression of the dysfunctional adaptation state of the subject and named this clinical semeiotic phenomenon functional dysmetria (FD).

On these premises, they developed the radio electric asymmetric conveyer (REAC) technology, a neuromodulation technology aimed at optimizing the best neuro-psycho-motor strategies in relation to environmental interaction. Neuro postural optimization (NPO) is a neurobiological stimulation treatment administered with the REAC technology and it has been specifically studied to treat the state of dysfunctional adaptation that is revealed through the presence of FD.

Aim

The purpose of this study was to verify whether a single administration of the REAC NPO treatment can trigger the improvement of the capacity of stress management and the quality of life in a population of children housed in a group home in Macapá, Brazil.

Materials and methods

The sample of this study consisted of nine children (six boys and three girls) in the age group of 6-11 years, which represented the totality of the children present in the structure. The children was investigated for the assessment of the presence of functional dysmetria and with the Pediatric Quality of Life Inventory TM 4.0 (PedsQL) before and one week after the administration of the REAC NPO.

Results

The stable disappearance of FD was found in all children at follow-up. In addition, improvements were found in stress management and quality of life, in the physical, emotional, social, and scholastic aspects evaluated with PedsQL.

Conclusions

It was seen that the REAC NPO neurobiological modulation treatment induced the stable disappearance of FD and triggered the initial improvement of neurophysical aspects also in a population of children housed in a group home in the Amazon region of Macapá, Brazil.

## Introduction

The imperfect symmetry of the body is a common and easily observable phenomenon, scientifically defined as fluctuating asymmetry (FA), and it is an expression of epigenetic stress [1] detected even in healthy subjects. The interaction of the organism and, therefore, of the nervous system, with environmental stressors, represents the origin of epigenetic stress and FA. In fact, environmental stress induces adaptive behaviors in the nervous system, which are expressed not only at the emotional, affective, cognitive, and relational levels, but also at the level of motor behavior. Motor behavior in relation to adaptive responses [2] causes the body to offer a smaller attack surface. To accomplish this, it generates an asymmetrical activation of symmetrical muscle groups. This phenomenon has been termed functional dysmetria (FD) [3,4] and is detectable even in healthy subjects [5].

To try to correct the FD, a neuromodulation procedure with radio electric asymmetric conveyer (REAC) technology called neuro postural optimization (NPO) was developed. NPO has demonstrated the capacity to remodel brain activities and a stable efficacy in the disappearance of FD [5]. In addition to these effects, previous studies showed that NPO can contribute to the effectiveness of other REAC neuromodulation treatments [6-9].

The purpose of this study was to verify that in a particularly fragile population, such as children housed in a group home, with family and social problems, the single administration of REAC NPO can trigger the initial improvement of stress management and quality of life in the physical, emotional, social, and scholastic aspects.

## Materials and methods

Ethics

This study was approved by the Research Ethics Committee of the Federal University of Amapá, Macapá, Amapá, Brazil (approval number N 4,855,825). This work is in line with the university's institutional objective of extending its activities beyond its walls, bringing an important technological contribution in health, and social support to the community. It should be noted that this study has also received authorization from the Judge of the Childhood and Youth Court, the president of the Foundation for Children and Adolescents of the State of Amapá, and the Technical Manager of the Casa Lar Ciã Katuá Institution, where the study was carried out.

This research was conducted according to the guidelines of the Declaration of Helsinki.

Population

Due to the high turnover of children admitted to the group home, convenience sampling was adopted for the study, to cover all children admitted during the data collection period who met the research criteria.

Children aged between six years and 11 years and 11 months, of both genders, who had been hosted for at least one week at the Abrigo Casa Lar Ciã Katuá in Macapá, Brazil, were considered eligible for the study. The enrollment for the study excluded the children who came to the group family after the start of the REAC NPO treatment applications, children in an acute phase of infectious disease, children who refused to participate in the study, children with a mental disorder or with poor cognitive level and non-cooperative behavior that would have interfered with the implementation of the procedures envisaged by the study.

Study design

This is an interventional open-label study.

Study timing

At T0, the children were administered the FD assessment and the Pediatric Quality of Life Inventory TM 4.0 (PedsQL) quality of life questionnaire. At T1, the NPO treatment was administered. At T2, immediately after the NPO administration, the FD assessment was repeated to check the positive response of the subject to the treatment, observable through the disappearance of the FD. At T3, one week after the NPO administration, the children were administered the FD assessment and the PedsQL quality of life questionnaire.

Assessment tests

The children were assessed for the detection of the FD and for the evaluation of their quality of life.

As per standardized procedure, FD was evidenced in children by asymmetrical activation of the contralateral quadriceps femoris and consequently by misalignment of the contralateral patella during the transition from supine to sitting position. The misalignment of the contralateral patellae was measured with a specific caliber called the dismetrometer. The dismetrometer (ASMED SRL, Florence, Italy), an instrument specifically designed for the assessment of interpatellar misalignment, was used to measure the FD.

The PedsQL quality of life questionnaire, validated in 2001 by Varni, Seid, and Kurtin [10] with its Brazilian version validated by Klatchoian [11], was administered to evaluate four parameters of quality of life: physical aspect, emotional aspect, social aspect, and school aspect. In this research, the school activity was evaluated within the questionnaire through the activities performed in the group home with the educators.

REAC neuro postural optimization (NPO) treatment

The REAC NPO is a neuromodulation treatment, consisting of a preprogrammed single session of a few milliseconds. It is administered by applying the tip of the metallic REAC asymmetric conveyer probe (ACP) to a specific area of the ear pavilion. The REAC medical device used in this study was the BENE mod. 110 (ASMED SRL, Florence, Italy).

Statistical analysis

The quantitative data on FD parameters and the PedsQL questionnaire were tabulated in a Microsoft Excel® spreadsheet (Microsoft Corporation, Redmond, Washington, United States), using the XRealStats supplement for the normality test and parametric and non-parametric statistical tests.

Descriptive statistics were also used to characterize the sample (absolute frequency, mean and standard deviation). The normal distribution of continuous variables before and after was tested using the Shapiro-Wilk test. For group comparisons at the time of entry, Student's parametric T-test was used for paired samples for normal variables, and for variables without normal distribution, Wilcoxon's non-parametric test was used. The significance level adopted was 0.05 for both tests.

## Results

**Figure 1:**
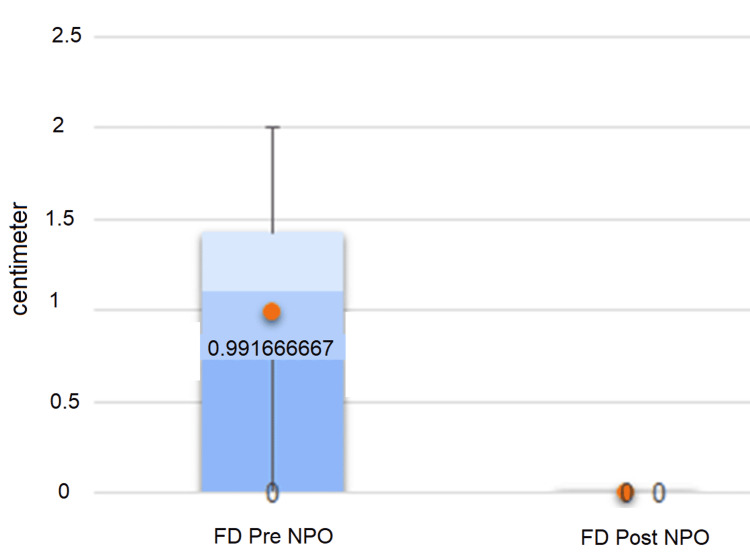
Average of FD values before and after NPO treatment. NPO: neuro postural optimization; FD: functional dysmetria

At T3, one week after the NPO administration, all the children showed the stable disappearance of the FD (Figure 1), with a statistical difference between the indices (t(11)= 5.17; p < 0.001). 

For the domains of the PedsQL instrument that presented normal distribution, namely emotional and school aspects, the paired Student's t-test was used, and for the physical and social aspects, the Wilcoxon test was used. The tests showed that there was a statistically significant difference in all domains, as shown in Table 1.

**Table 1:** Relationship between the means and medians of the PedsQL questionnaire variables before and after the NPO treatment. * The values of the physical and social aspects before and after therapy correspond to the median PedsQL: Pediatric Quality of Life Inventory TM 4.0; NPO: neuro postural optimization

Variables	T0 PedsQL	T3 PedsQL	P-value	Z or t value
Physical Aspect*	68.75	87.5	0.003	z = 2.1
Emotional aspect	34.44	53.88	0.004	t (8) = -3.97
Social aspect*	60	75	0.01	z = 2.30
School aspect	53.88	66.66	0.04	t (8) = -2.36

## Discussion

Although the group home represents a welcoming place and provides children with certain well-being, studies reveal that the children housed in group home experience more stressful events when compared to children who live with their families [12]. Moreover, the children who experience psychosocial adversities that lead them to be housed in a group home are at high risk of being affected by negative repercussions on mental health [13].

Previous studies have shown that REAC neurobiological treatments are effective in optimizing the individual response to environmental stressors, even in children in a situation of severe socioeconomic and cultural suffering [6]. In this study, the REAC NPO treatment was seen to be able to induce the stable disappearance of the FD in all the children treated. This result can be explained as a remodulation of brain activity in the sense of greater efficiency and competence of the activated areas [4, 5].

As regards the quality-of-life indexes, the NPO treatment was able to trigger the improvement of the emotional aspect of the PedsQL questionnaire, which addresses questions related to negative feelings such as sadness, anger, and worry. As for the social parameter score, the post-NPO treatment results suggest a decrease in difficulties related to socialization. Regarding the school aspect of PedsQL, children were not attending school during the data collection period because of the coronavirus disease 2019 (COVID-19) pandemic. However, they performed daily activities such as painting, drawing, writing, and reading with assistance from the group home’s educators and caregivers. Even in the face of this obstacle in school development, there was a slight improvement in the difficulties present in the questionnaire, such as: paying attention in class, forgetting what you learned, and accompanying colleagues in activities.

These results confirmed the improvement observed in a previous study about school performance and communicative ability, such as the improved pronunciation of words, in children of a family with severe socioeconomic and cultural hardship [6].

Study's limitations

The intrinsic limits of this study are that of being open-label and the fact that the sample while representing 100% of the subjects present in the structure, is small. Considering that the REAC NPO treatment used in this study has been used and studied in all its aspects for years, the initial limitations take on a different meaning. The results of this study, therefore, represent further confirmation of what was previously demonstrated, even in a particularly fragile population.

## Conclusions

The single administration of the REAC NPO treatment enabled FD recovery and was able to promote the initial improvement of quality of life, scholastic and socialization skills, and the overall state of physical and mental health of children in the age group of 6-11 years housed in a group home. This result is particularly significant considering that there was no change in the stressful environment the children lived in.

Even though further studies on a more robust sample size would be appropriate, the evidence of this work suggests that the REAC NPO treatment, due to its non-invasive characteristics, painlessness, and speed of administration, can be a very useful therapeutic tool in support of fragile individuals who experience very stressful environmental interaction that can trigger dysfunctional adaptive behaviors at the origin of disorders and diseases.
